# An Efficient Composite Modifier Prepared for Enhancing the Crystallization and Flame-Retardancy of Poly(m-xylylene adipamide)

**DOI:** 10.3390/polym14173626

**Published:** 2022-09-01

**Authors:** Zhifeng Zhao, Yueyang Tan, Shangzhen Guo, Xiuyuan Ni

**Affiliations:** State Key Laboratory of Molecular Engineering of Polymers, Department of Macromolecular Science, Fudan University, Shanghai 200438, China

**Keywords:** poly(m-xylylene adipamide), nucleating agent, composite, crystallization, flame-retardancy

## Abstract

Poly(m-xylylene adipamide) (MXD6) has good gas barrier properties and high mechanical strength. However, in nature, this resin has a low rate of crystallization. In order to overcome this obstacle in its applications, this study prepares a new, efficient modifier for MXD6 by combining the synthesized DOPO derivative (DT) and P22. It is found that the use of the binary modifier exhibits obvious effects on the crystallization of MXD6. When 11.0 wt.% DT is added together with 0.1 wt.% P22 (DT/P22), the crystallization temperature of MXD6 shifts to a higher temperature of 19.7 °C, and the crystallinity degree of MXD6 is significantly increased by 60%. Meanwhile, this modifier exhibits obviously intumescent flame-retardancy on MXD6 by increasing the limited oxygen index (LOI) from 26.4% to 33.4%. The results of the cone calorimeter test (CCT) reveal that the peak heat release rate (PHRR), total heat release (THR) and average effective heat release (av-EHC) are obviously suppressed due to the use of this modifier. Moreover, the influences of this modifier on the crystal structures, mechanical and rheological properties of MXD6 are analyzed in detail. This study can provide an efficient modifier for MXD6.

## 1. Introduction

Poly(m-xylylene adipamide) (MXD6), one of the crystalline polyamides, is produced through polycondensation of a meta-xylene diamine with adipic acid [1]. With typical triclinic crystalline [2], MXD6 is the only semi-aromatic polyamide containing m-xylene groups [3]. MXD6 has better mechanical strength and thermal stability than PA6 [4,5,6]. The fact that MXD6 has a melting temperature of 237 °C allows it to be relatively easily melted and molded among the semi-aromatic polyamides. As reported, MXD6 has been used for plastic parts in the fields of electronic, electrical and automotive industries, etc. [7,8,9,10]. In particular, MXD6 can exhibit excellent barrier effects to various gases, including O_2_ and CO_2_, and the gas resistance can be maintained within a broad range of humidity. With the strong gas-barrier effects, MXD6 is an ideal alternative to packaging materials, which have growing applications in the field of medicine and food [11,12,13,14,15,16,17].

The properties of plastic materials are directly related to the crystalline morphology and crystallization behaviors during processing [18]. However, the crystallization rate of MXD6 is relatively slow [18,19,20,21,22] due to the steric hindrance of the benzene ring in its structure [23]. In addition, as a result of the low crystallinity degree, a subsequent-crystallization usually takes place, which should be avoided in injection molding [21]. Therefore, the crystallization behavior for MXD6 strongly influences both barrier properties and processability [11], which is a key for developing new MXD6 materials.

There have been studies on the crystallinity of MXD6 by using various nanoparticles as the nucleating agents, which include montmorillonites, kaolinite, polyhedral oligomeric silsesquioxane (POSS) and carbon nanotubes [24,25,26,27]. Based on these reports, the crystallinity degree of the MXD6 resin is increased from 20% to 42%, and the crystallization temperature shifts to high temperatures from 5 °C to 13 °C, depending on the nature of the nanoparticles used. However, a composite of montmorillonite and POSS was reported to show lower improvement than each of the components [27]. In addition, the crystallization kinetics of MXD6 were investigated by using the blend of PA66 and talc as a nucleating modifier, and the shear-induced crystallization was reported [18,20,28].

In general, plastics should reach flame retardancy standards, which establish them as safe for users. However, MXD6 has a limit oxygen index (LOI) of about 26%, lower than the typical requirement. It is observed that during the combustion process, MXD6 shows obvious melt drips due to a low melt viscosity, which leads to the fast spread of fire. This property is similar to most thermoplastics [29]. Thus, it is necessary to raise the flame retardancy of MXD6 to ensure its applications, especially in the electronical, aircraft and automobile fields [30,31,32]. The commonly used flame retardants in plastics are classified into organic retardants [33,34] and inorganic retardants [35,36], which include metal hydroxides, mineral fillers and aluminum hypophosphite. As for the inorganic retardants, a large amount of the retardants is always added to reach a considerable level of retardancy. However, at high contents of inorganic modifiers, these plastics usually undergo undesirable changes in their properties, including mechanical performance.

In this work, we carried out the studies of modifying the crystallization and flame retardancy of MXD6 and obtain a new composite modifier that can greatly enhance the crystallizing ability and flame retardancy of MXD6 simultaneously. This modifier is composed of the phosphorus nitrogen intumescent flame retardant (DT) and nucleating agent. A synergistic effect on the crystallization is measured. We synthesize DT by the addition reaction of 9,10-dihydro-9-oxa-10-phosphaphenanthrene-10-oxide (DOPO) and 2,4,6-triallyloxy-1,3,5-triazine (TAC) [37]. The organic DT flame retardant is meltable and easy to mix with the polymers. Moreover, DT shows a high thermal stability at the initial degradation temperature of 340 °C. This temperature can meet the requirement for the melting process of MXD6 at temperatures of around 270 °C [38]. The non-isothermal and isothermal crystallization of the modified MXD6 are studied by differential scanning calorimetry (DSC) and polarized optical microscopy (POM). The flame-retardant properties have been investigated by the limited oxygen index (LOI) and cone calorimeter test (CCT). The influences of this modifier on the crystal structures and mechanical and rheological properties of MXD6 are also analyzed in detail.

## 2. Materials and Methods

### 2.1. Materials

**Scheme 1:**
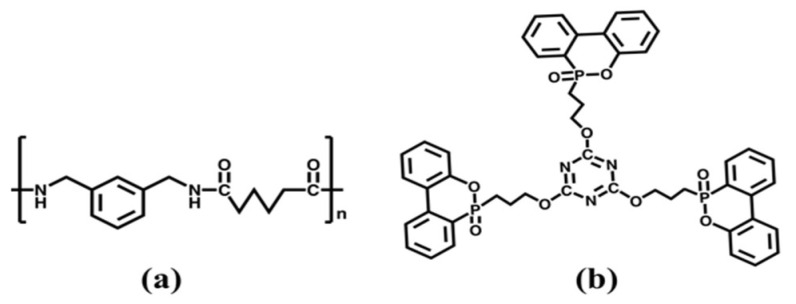
The chemical structure of (**a**) MXD6 and (**b**) DT.

MXD6 (number-average molecular weight is 16,000 g/mol; the relative viscosity is η_r_ = 2.1; the density is 1.21 g/cm^3^) was supplied by Sinochem Technology Co., Ltd. Shanghai, China. Nucleating agent Bruggolen P22 (>99%) was purchased from Brüggemann. The structures of MXD6 and DT are shown in Scheme 1.

### 2.2. Preparation of the Modified MXD6

MXD6 pellets were dried in a vacuum oven at 110 °C for 12 h. DT and P22 were dried in a vacuum oven at 80 °C for 12 h. The blends of MXD6 and the composite DT/P22 modifier were blended according to the formulation and were prepared using a fully intermeshing co-rotative twin-screw extruder (HAAKE Polylab OS, Thermo Fisher, Waltham, MA, USA, D = 16 mm, L/D = 25/1), and followed by rapid air cooling before being cut into pellets. The screw speed was 30 rpm, and operating temperatures from the hopper to die section were set at 220/250/250/260/260/260/265/265/265/240 °C, respectively. The extruded MXD6 pellets were dried in a vacuum oven at 80 °C for 24 h to remove moisture before being injected (at 270 °C) or hot-pressed (at 280 °C under 20 MPa for 5 min) into various specimens for tests. The contents of MXD6, DT and P22 in these blends are shown in Table 1. The synthesis steps of the DOPO-derived flame retardant (DT) were described in our previous work [37].

### 2.3. Characterization

#### 2.3.1. Differential Scanning Calorimetry (DSC)

Differential scanning calorimetry Q2000 DSC (TA Instruments, New Castle, DE, USA) was used under nitrogen stream, with the samples of 8–10 mg. Four scanning cycles were conducted at temperatures ranging from 40 to 300 °C. At the end of the first heating ramp, the samples were held at 300 °C for 10 min to erase the thermal history. The heating rate was set at 10 °C/min. The cooling rates were set at 10 and 40 °C/min for non-isothermal crystallization, respectively. Data were collected from the first cooling ramp. The degree of crystallinity (Xc) was calculated according to the following equation [39]:

(1) $$\mathrm{Crystallinity}\;(\%)=\frac{\Delta H_{c}}{\;\Delta H_{0}\left(1-x\right)}$$

where (1 − *x*) is the percentage of MXD6, and Δ*H_c_* is the enthalpy of crystallization obtained from the cooling scan. Δ*H*0 is the heat of fusion for 100% crystalline MXD6, which is 175 J/g [40].

#### 2.3.2. Polarized Optical Microscopy (POM)

A Leica DM2500P (Leica, Germany) polarizing microscope system, equipped with a Linkam hot stage (THMS600, Linkam, Salfords, UK), was used to measure the crystal morphologies of the neat MXD6 and the composites during the isothermal crystallization processes. 

#### 2.3.3. Limited Oxygen Index (LOI)

The LOI values were measured by using an XYC-75 oxygen index meter (Hebei, China) in the procedures according to ASTM D2863-97. For the neat MXD6 and modified MXD6 materials, each sample has dimensions of 130 × 6.5 × 3.2 mm, and the average values were calculated from the data of at least five samples tested.

#### 2.3.4. Cone Calorimeter Test (CCT)

The CCT tests were carried out on an FTT cone calorimeter (Fire Testing Technology, West Sussex, UK) according to ISO 5660-1 under a heating flux of 50 kW/m^2^. For the neat MXD6 and modified MXD6 materials, two samples with the dimensions of 100.0 × 100.0 × 3.0 mm, in the weight of about 31 g, were measured to acquire the normal CCT curves.

#### 2.3.5. Field Emission Scanning Electron Microscopy (FESEM)

The micro morphologies of residual chars after the CCT tests were recorded on a Zeiss Gemini SEM500 (Zeiss, Germany) under a high vacuum at an accelerating voltage of 3 kV.

#### 2.3.6. Rheological Properties

The rheological measurements of the melts were conducted by using a HAAKE Mars III Rheometer (Thermo Fisher, Waltham, MA, USA) with a plate–plate mode at N_2_ atmosphere and 250 °C. The tests were carried out at 1% strain within the frequency range of 0.06–100 rad/s. 

#### 2.3.7. Mechanical Properties

The tensile tests were conducted by a universal tensile testing machine (Instron-5966, Norwood, MA, USA) stretched at a rate of 20 mm/min, with the sample dimensions of 74 × 5 × 2 mm according to the ASTM standard D1708-02.

## 3. Results and Discussion

### 3.1. The Crystallizing Properties of the Modified MXD6

**Figure 1:**
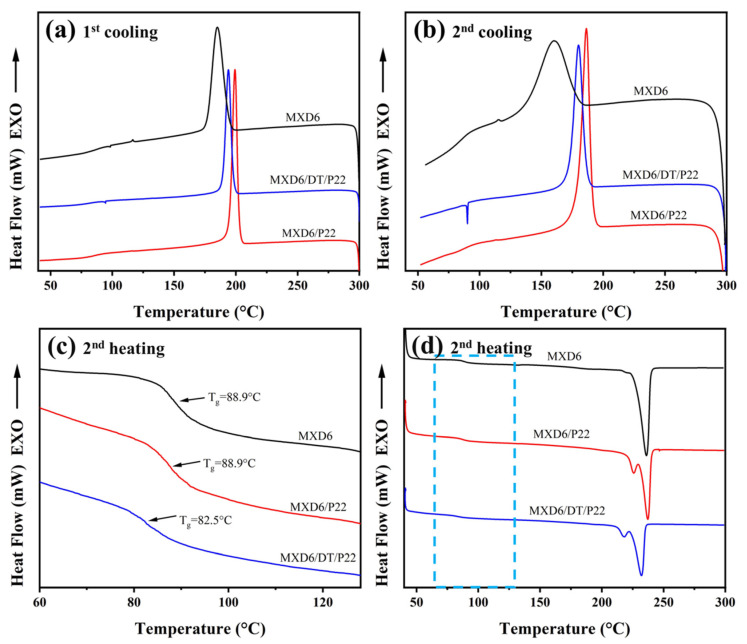
DSC curves of neat and modified MXD6: (**a**) first cooling (10 °C/min) ramp, (**b**) second cooling (40 °C/min) ramp and (**c**,**d**) second heating (10 °C/min) ramp.

Figure 1a compares the non-isothermal crystallization DSC curves of the neat MXD6 and the modified MXD6 materials at a low cooling rate of 10 °C/min. It is observed that the neat MXD6 can crystallize with a relatively broad exothermic peak appearing at 185.0 °C. When MXD6 is modified by adding P22 or the composite modifier, the samples of the two materials consistently exhibit very sharp crystallization peaks, and the two peaks obviously shift to higher temperatures. The narrower crystallization peaks indicate that the process of crystallization has been accelerated. The observation of this peak shift proves that the polymer can crystallize at higher temperatures. Therefore, it is concluded that the crystallization properties of MXD6 are significantly improved by the modifiers. Table 2 gives the crystallization parameters in detail, which include the onset temperatures of crystallization (To), crystallization temperatures (Tc, i.e., the peak temperature), ΔHc, and degree of crystallinity (Xc). The value of To-Tc is a measure of crystallization rate. It is obtained that when 11.0 wt.% DT together with 0.1 wt.% P22 (DT/P22) is used, Tc shifts to a higher temperature by 9 °C, the value of To-Tc is decreased from 15.6 to 9.4 °C, and Xc is increased by 20.1%. Furthermore, we carried out the non-isothermal crystallization of the neat MXD6 and the modified MXD6 at 40 °C/min, which is approximate to the rapid cooling rates in industrial plastics processing. The DSC curves and the crystallization parameters at 40 °C/min are shown in Figure 1b and Table 2, respectively. The results show that when 11.0 wt.% DT together with 0.1 wt.% P22 (DT/P22) is added, Tc shifts to a higher temperature of 19.7 °C, and the value of To-Tc is decreased from 25.9 to 13.6 °C. Xc is increased by 60.4%. Conclusively, the above results prove that the crystallization ability of MXD6 can be greatly improved by the composite modifier consisting of the nucleating agent P22 and the synthesized flame-retardant DT.

As indicated by the data in Table 2, when only nucleating agent P22 is added in MXD6, Xc is increased by 10.8% and 46.7% at 10 and 40 °C/min, respectively. It is important to find that when the composite of P22 and DT is used, Xc is increased by 20.1% and 60.4% at 10 and 40 °C/min, respectively. This result strongly demonstrates that the DT can assist the nucleating agent in the aspect of the degree of crystallinity, meaning a synergistic effect. DT has a function of lubrication or plasticization, which makes the melt viscosity of MXD6 lower. Under this condition, the addition of P22 provides numerous crystal sites, which speed up the crystallization of MXD6.

Figure 1c,d shows the second heating DSC ramps of the neat and modified MXD6 at a heating rate of 10 °C/min. The measured glass transition temperatures (Tg) and melting temperatures (Tm) are presented in Table 3. The neat MXD6 displays the Tg at 88.9 °C and Tm at 236.2 °C. The addition of P22 has not changed the Tg distinctly; meanwhile, a new shoulder endothermic peak appears prior to the chief endothermic peak, which retains the same position. This peak is assigned to the primary crystallization due to the heterogeneous nucleation of the used P22 [40]. When the DT/P22 composite is added, the Tg and Tm shift to a lower temperature from 7.4 and 4.1 °C, respectively. This result can be ascribed to the lubrication or plasticization of the organic DT [37,41].

**Figure 2:**
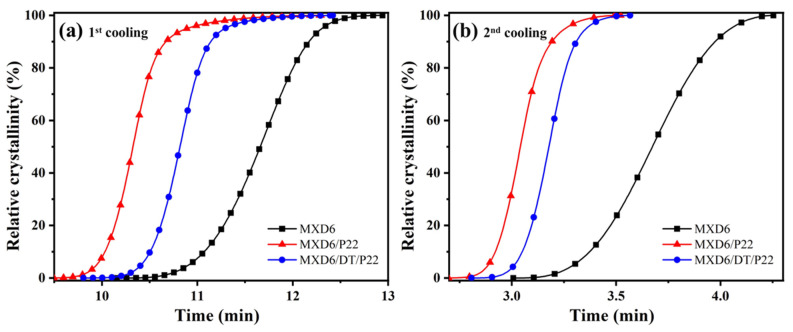
Relative crystallinity vs. time at different cooling rates: (**a**) 10 °C/min and (**b**) 40 °C/min of neat and modified MXD6.

The relative crystallinity *X*(*t*) at different times is calculated with the following equation [42].

(2) $$X\left(t\right)=\frac{X_{c}\left(t\right)}{X_{c}\left(t_{\infty }\right)}=\frac{\int_{t_{0}}^{t}\left(\frac{dH}{dt}\right)dt}{\int_{t_{0}}^{t_{\infty }}\left(\frac{dH}{dt}\right)dt}=\frac{\Delta H_{t}}{\Delta H_{t_{\infty }}}$$

where $t_{0}$ and $t_{\infty }$ are the onset and infinite time of the crystallization, respectively. $\Delta H_{t}$ and $\Delta H_{t_{\infty }}$ are the enthalpy at time t and the total enthalpy at the end of crystallization, respectively. *dH*/*dt* is the heat flow rate. The calculated values of *X*(*t*) at different times are depicted in Figure 2.

It can be clearly seen in this figure that, under the same cooling rate, MXD6 takes a much longer time to complete the crystallization than the other two samples containing P22 and the DT/P22 composite, respectively. This result confirms that the use of the DT/P22 composite can obviously accelerate the crystallization of MXD6. The *X*(*t*) curves can be divided into three sections: nucleation in the range of low crystallinity around several percent, followed by the crystal growing section and the constant section. As indicated in Figure 2, compared to the MXD6 sample containing only P22, the MXD6 modified by DT/P22 composite nucleates relatively slowly, but the two samples have almost the same crystal growing rates according to the slopes of the *X*(*t*) curves. This result illustrates that P22 possesses a strong heterogeneous nucleation effect, and the coexistence of DT has not discernibly interfered with the crystal growth at the same cooling rate. Importantly, it is clearly obtained from Figure 2 that the use of the DT/P22 composite modifier has enhanced the nucleation and crystal growth compared with the neat MXD6. 

### 3.2. The Crystalline Morphologies

**Figure 3:**
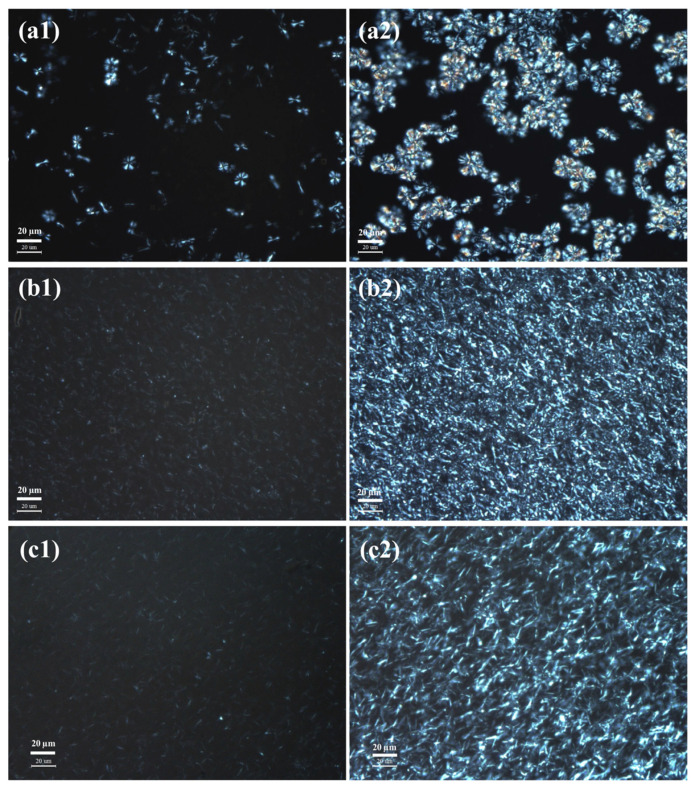
The POM pictures of isothermal crystallization of (**a1**,**a2**) MXD6, (**b1**,**b2**) MXD6/P22, (**c1**,**c2**) MXD6/DT/P22 at 216, 220 and 213 °C at 5 and 10 min, respectively.

The crystal patterns of the neat MXD6 and the modified MXD6 were detected by POM to disclose the effects of the DT/P22 modifier. It is observed that when either the P22 or DT/P22 modifiers are used, the crystals grow rapidly, leading to high packing density. Figure 3 shows the POM pictures that are taken at 5 and 10 min after the samples reach the set temperatures, respectively. The clear patterns of the nucleus are seen in the pictures that are taken at 5 min. As seen, the neat MXD6 tends to form spherical crystals with the maltese-cross patterns. As for the MXD6/P22 and MXD6/DT/P22, they tend to form new crystals in the form of a “rod” shape with a large aspect ratio. Conclusively, the use of the DT/P22 composite modifier can change the crystal morphologies of MXD6. It has been demonstrated in lots of relative studies that the mechanical properties of crystalline polymers are associated with their crystal shapes, such as polypropylene [43], and the crystals in the form of sheets or shish-kebab would favor the mechanical performance. The changes in the crystal shapes of MXD6 probably have similar influences on its properties.

### 3.3. The Flame-Retardancy of Modified MXD6 Material

#### 3.3.1. LOI and Cone Calorimeter Tests

**Figure 4:**
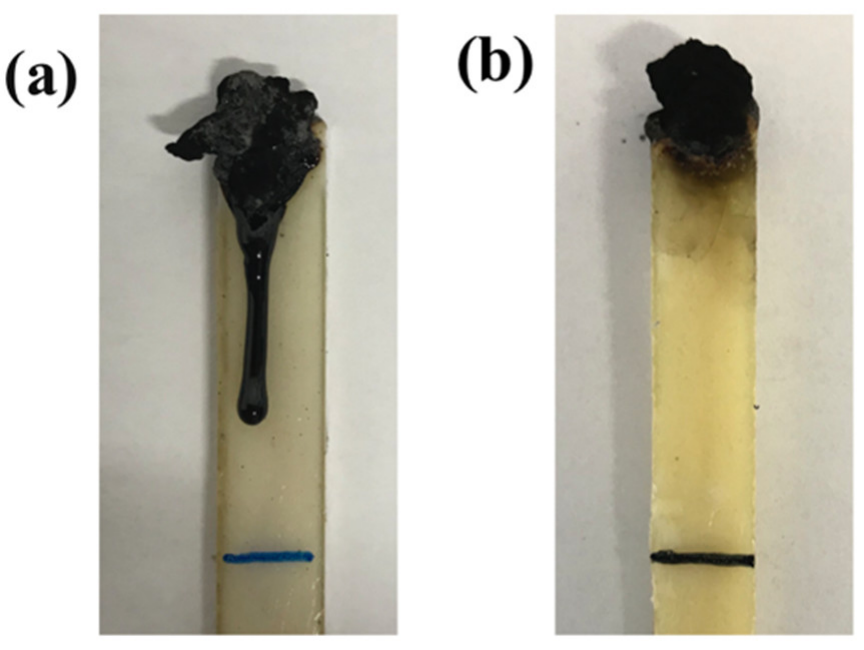
The digital images after the LOI test: (**a**) MXD6 and (**b**) MXD6/DT/P22.

Here, we evaluate the flame retardancy of the neat MXD6 and the modified MXD6 by LOI and CCT tests. Figure 4 shows the digital images of the material strips that remained after the LOI tests. It is measured that the neat MXD6 has an LOI value of 26.4%, less than the level ruled for the flame-retardant materials (>28%). These data indicate that the flame retardancy of MXD6 must be improved before it is used in practical applications. In addition, there is a heavy melt-dripping for the neat MXD6 during combustion, as shown in Figure 4a. When 11.0 wt.% DT together with 0.1 wt.% P22 (DT/P22) is added to MXD6, the LOI value of this modified MXD6 is 33.4%, meaning excellent flame retardancy. It is also important that the original melt-dripping is nearly completely suppressed in the modified MXD6, as shown in Figure 4b. The large improvement in the flame retardancy is attributed to the synthesized DT component here because the content of P22 is too low at 0.1 wt.%.

**Figure 5:**
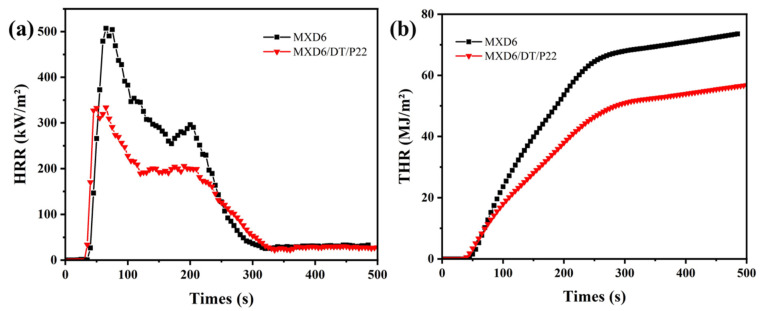
(**a**) HRR and (**b**) THR curves of neat MXD6 and MXD6/DT/P22 during the CCT test.

CCT is an effective method for simulating the combustion of materials in real fire scenarios under laboratory conditions [44]. Figure 5 presents the CCT curves under a heating flux of 50 kW/m^2^. Table 4 lists the obtained values of the heat release rate (HRR), total heat release (THR) and time to ignite (TTI). As observed, the neat MXD6 has a PHRR of 507.2 kW/m^2^, and the modified MXD6 has a PHRR of 333.8 kW/m^2^, which is 34.2% lower than that of neat MXD6. Moreover, the THR of the modified MXD6 is decreased by 21.2% compared to neat MXD6.

The Flame Retardancy Index (FRI) is used to evaluate the overall flame retardancy [45]. The FRI is defined as the ratio of $\mathrm{THR}*\left(\frac{\mathrm{PHRR}}{\mathrm{TTI}}\right)$ between the neat MXD6 and the corresponding flame-retardant blends:

(3) $$\;\mathrm{FRI}=\frac{{\left[THR*\left(\frac{PHRR}{TTI}\right)\right]}_{neat\;MXD6}}{{\left[THR*\left(\frac{PHRR}{TTI}\right)\right]}_{FR\;\;MXD6}}$$

where *PHRR*, *THR*, and *TTI* are the results in Table 4. The FRI can be categorized into <1, 1–10 and 10–100, corresponding to the flame retardancy performance symbolized as “Poor”, “Good” and “Excellent”, respectively. In this study, the incorporation of DT/P22 makes the FRI of the composites equal to 1.65, which means MXD6/DT/P22 has a “good” flame retardancy performance compared to neat MXD6. We found the CCT data on two important polyamide materials (PA66 and PA6) that used DOPO-based compounds as flame retardants. Their DOPO-based compounds have different structures from that of our study. The FRI value of the PA66 material is 1.26 [37], and the PA6 material is 1.31 [46]. Their FRI values are lower than that of our study (1.65).

**Figure 6:**
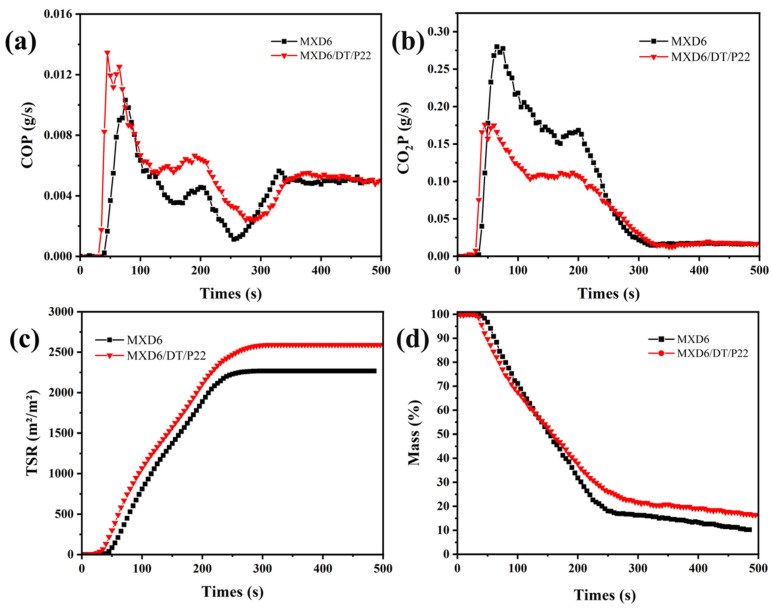
Rate of (**a**) CO release, (**b**) CO_2_ release, (**c**) total smoke release and (**d**) mass loss curves during combustion in the CCT test of neat MXD6 and MXD6/DT/P22.

The effective heat of combustion (EHC), which is defined as the ratio of HRR to the mass loss rate, represents the combustion behavior of volatile gases [47]. The average effective heat of combustion (av-EHC) is shown in Table 4. When the composite modifier is added in MXD6, the value of the av-EHC is decreased by 14.8%, indicating insufficient combustion. Figure 6 shows the release of carbon monoxide (CO), carbon dioxide (CO_2_), total smoke release (TSR) and mass loss curves during combustion. The corresponding data are given in Table 5. The CO and CO_2_ release of the MXD6/DT/P22 are increased by 49.1% and decreased by 18.9% compared with neat MXD6, respectively. When the DT/P22 composite modifier is added, the ratio of CO/CO_2_ is enhanced from 0.048 to 0.087 by the degree of 81.3%. Moreover, the value of TSR increased from 2266.4 to 2571.2 m^2^/m^2^ (Figure 6a–c). After the combustion, the amount of the final combustion residues is increased from 9.2% to 15.1% due to the use of the composite modifier. It is 64.1% higher than that of neat MXD6. The high content of residue contributes to the condensed phase flame retardancy performance. As an organic phosphorus flame retardant, the thermal cleavage of DT can yield the phosphorus-contained radicals such as PO• and PO_2_• during combustion [37]. The coupling reactions will take place between these phosphorus-contained radicals and the combustion-responsible radicals in the gas phase, such as •OH [37]. The termination reactions of these radicals can inhibit the burning reactions, which includes the delay in converting CO to CO_2_. This gas-phase flame retardancy is supported by insufficient combustion as measured, with the CO_2_ release decreasing and both total smoke release and the burned residues increasing.

#### 3.3.2. Analysis of Char Residues

**Figure 7:**
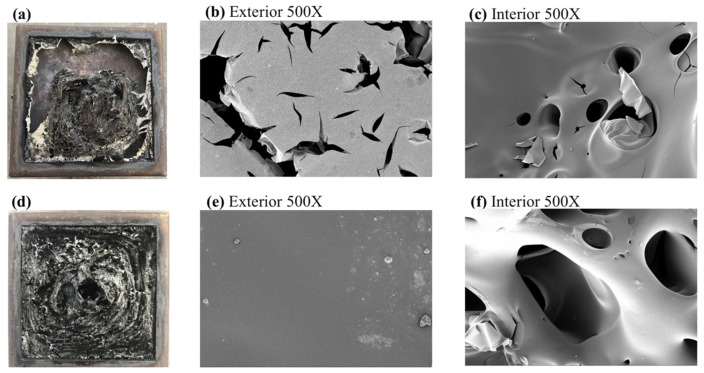
Digital and FESEM images of CCT residual chars, (**a**–**c**) neat MXD6 and (**d**–**f**) MXD6/DT/P22.

Figure 7 shows the digital and FESEM images of CCT residual chars. A large number of cracks and holes exist in the external surface of the neat MXD6 char layer (Figure 7b). The modified MXD6 exhibits a more compact and coherent carbon layer (Figure 7e). The compact carbon layer should be promoted by the involatile phosphorus residues of DT during combustion [37]. This compact layer covering the surface can slow down the heat and mass transfer between the underside polymers and the burning gases. It can protect the substrate from rapid decomposition and can also reduce the emissions of the combustible gases from the interior into burning gases [37]. This condensed-phase flame retardancy is supported by the interior structure of the burned sample (Figure 7f). As seen, more and larger honeycombs are formed in the modified MXD6 than the neat MXD6. Clearly, the interior gases, which can be produced from DT [37] and chains, have to extend in the bulk of the modified MXD6 due to the shield of the compact layer. With the FESEM and early CCT results, DT should exhibit the flame retardancy in both the gas and condensed phases.

### 3.4. Rheological and Mechanical Properties

**Figure 8:**
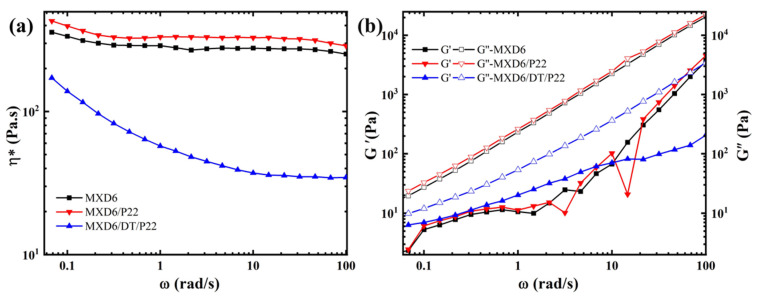
(**a**) Complex viscosity (η*), (**b**) storage modulus (G′) and loss modulus (G″) vs. frequency of neat and modified MXD6 at 250 °C.

Figure 8 shows the complex viscosities (η*), storage modulus (G′) and loss modulus (G″) of the samples as a function of angular frequency at 250 °C. When DT/P22 composite is added, the modified MXD6 has a low complex viscosity [48] and shows obvious “shear thinning” behavior as compared to neat MXD6 (Figure 8a). As shown in Figure 8b, the G″ of all samples is greater than their respective G′. In the low-frequency region (ω < 10 rad/s), the G′ of MXD6/DT/P22 is higher than that of neat MXD6 but significantly lower than neat MXD6 at higher frequency (ω > 10 rad/s). DT is a polar polymer of low molecular weight. There should be strong interactions between DT and the amide groups in MXD6 chains [37]. In the melt blend, the intramolecular interactions among the MXD6 chains are weakened due to the presence of DT, and the entanglement of MXD6 chains also turns weak. Due to this plasticization effect, the viscosity of MXD6 melt drops obviously, and the melt blend has different rheological properties from the neat MXD6 resin.

Table 6 lists the tensile strength, elongation at break and modulus of the neat MXD6 and the MXD6/DT/P22 materials. As seen, the DT/P22 MXD6 shows good overall mechanical properties, in addition to the excellent flame retardancy as described before. In particular, when the DT/P22 composite modifier is added, the elastic modulus of MXD6 is significantly enhanced. This result is attributed to the joint effects of DT and P22. DT acts as the plasticizer due to the interactions with MXD6 chains, being capable of enhancing the material’s stretchability. Meanwhile, this material can be strengthened by P22 by increasing the degree of crystallization and changing the crystal shape.

## 4. Conclusions

In this work, a new and efficient composite modifier has been successfully prepared to modify poly(m-xylylene adipamide) (MXD6). These modifiers that combine the synthesized DOPO derivative (DT) and P22 have obvious effects on the crystallization, flame retardancy and processing properties of MXD6. When 11.0 wt.% DT together with 0.1 wt.% P22 is added, the crystallization temperature of MXD6 shifts to a higher temperature of 19.7 °C, and the crystallinity is greatly increased by 60%. Meanwhile, the LOI value is enhanced from 26.4% to 33.4%. When this modifier is used, the crystals of MXD6 tend to transform from spherical crystals to a “rod” structure. As compared to the neat P22 nucleating agent, the coexistence of DT increases the crystallization degree of MXD6.

This modifier exhibits good flame retardancy on MXD6. It can decrease the peak heat release rate, total heat release, average effective heat release and carbon dioxide yield of MXD6 by 34.2%, 21.2%, 14.8% and 18.9%, respectively. Moreover, the yield of carbon monoxide and residue is increased by 49.1% and 64.1%, respectively. This study can obtain an efficient modifier for MXD6 in both crystallization and flame retardancy.

## Figures and Tables

**Table 1:** The formulation of modified MXD6.

Samples	MXD6 (wt.%)	DT (wt.%)	P22 (wt.%)
MXD6	100.0	0	0
MXD6/P22	99.9	0	0.1
MXD6/DT/P22	88.9	11.0	0.1

**Table 2:** The non-isothermal crystallization results of neat and modified MXD6.

Samples	Cooling Rate (°C/min)	To (°C)	Tc (°C)	ΔHc (J/g)	Xc (%)
MXD6	10	200.6	185.0	46.9	26.8
	40	186.2	160.3	31.8	18.2
MXD6/P22	10	208.6	199.3	51.9	29.7
	40	199.8	186.3	46.7	26.7
MXD6/DT/P22	10	203.4	194.0	50.1	32.2
	40	193.6	180.0	45.4	29.2

**Table 3:** DSC results of the 2nd heating process of neat and modified MXD6.

Samples	Tg (°C)	Tm (°C)
MXD6	88.9	236.2
MXD6/P22	87.6	237.2
MXD6/DT/P22	82.5	232.1

**Table 4:** The LOI and CCT data of neat MXD6 and MXD6/DT/P22.

Samples	TTI (s)	PHRR (kW/m^2^)	THR (MJ/m^2^)	av-EHC (kJ/g)	LOI (%)
MXD6	34	507.2	73.5	23.6	26.4 ± 0.2
MXD6/DT/P22	29	333.8	57.9	20.1	33.4 ± 0.3

**Table 5:** Parameters during combustion of neat MXD6 and MXD6/DT/P22.

Samples	TSR (m^2^/m^2^)	av-SEA (m^2^/kg)	av-COY (g/kg)	av-CO_2_Y (g/kg)	CO/CO_2_ Yield Ratio	Residue (%)
MXD6	2266.4	703.8	73.5	1544.0	0.048	9.2
MXD6/DT/P22	2571.2	877.1	109.6	1252.9	0.087	15.1

**Table 6:** The mechanical properties of neat and modified MXD6.

Samples	Tensile Strength (MPa)	Elongation at Break (%)	Modulus (MPa)
MXD6	80.6 ± 8.3	6.4 ± 0.7	1734.0 ± 61.3
MXD6/P22	91.8 ± 10.4	5.6 ± 0.9	1825.0 ± 53.8
MXD6/DT/P22	79.2 ± 11.8	4.4 ± 0.8	2075.6 ± 41.1

## Data Availability

Not applicable.
